# Caveolin-initiated macropinocytosis is required for efficient silica nanoparticles’ transcytosis across the alveolar epithelial barrier

**DOI:** 10.1038/s41598-022-13388-7

**Published:** 2022-06-08

**Authors:** Pascal Detampel, Sara Tehranian, Priyanka Mukherjee, Morgan Foret, Tobias Fuerstenhaupt, Ali Darbandi, Nawaf Bogari, Magda Hlasny, Ayodeji Jeje, Michal A. Olszewski, Anutosh Ganguly, Matthias Amrein

**Affiliations:** 1 Department of Cell Biology and Anatomy, University of Calgary, Calgary, AB Canada; 2 Department of Chemical and Petroleum Engineering, University of Calgary, Calgary, AB Canada; 3 Department of Microbiology Immunology and Infectious Diseases, University of Calgary, Cumming School of Medicine, 3330 Hospital Drive, Calgary, AB T2N4N1 Canada; 4 Division of Pulmonary and Critical Care Medicine, Department of Internal Medicine, University of Michigan Health System, Ann Arbor, MI USA; 5 Research Service, LTC Charles S. Kettles VA Medical Center, Ann Arbor, MI USA; 6 Department of Surgery, Michigan Medicine, University of Michigan, Ann Arbor, MI USA; 7 Present Address: Division of Pharmaceutical Technology, Department of Pharmaceutical Sciences, University of Basel, Basel, Switzerland

**Keywords:** Cell biology, Respiration

## Abstract

Removal of particulate materials that would otherwise cumulate within the airspace and hinder the gas exchange is one of the central processes of maintaining lung homeostasis. While the importance of the particle uptake by alveolar macrophages and their expulsion via the airways mucociliary escalator is well established, very little is known about the alternative route for removing the particles via direct crossing the lung epithelium for transfer into the pulmonary lymph and bloodstream. This study dissected sequential mechanisms involved in nanoparticle transcytosis through the alveolar epithelial cell layer. By a combination of live cell, super resolution, and electron microscopy and RNA interference study, we have dissected temporal steps of nanoparticle transcytosis through alveolar epithelium. Our study revealed that caveolin is essential for the firm adhesion of the silica nanoparticle agglomerates to the apical membrane and their subsequent rapid internalization with the help of macropinocytic elements C-terminal-binding protein1 and Rabankyrin-5 but not dynamin. Actin, but not microtubules, played a major role in nanoparticle uptake and subsequent transportation. The compartments with nanoparticles were tethered to trans-Golgi network to be jointly transported along actin stress fibers across the cytoplasm, employing a myosin-dependent mechanism. The trans-Golgi nanoparticle transport machinery was positive to Rab6A, a marker linked to vesicle exocytosis. Exocytosis was primarily occurring at the basolateral plane of the alveolar epithelial cells. The high-proficiency novel caveolin and Rabankyrin-5 associated uptake and transcellular transport of nanoparticles across the AEC barrier supports its importance in clearance of amorphous silica and other types of non-inflammatory nanoparticles that are rapidly removed from the lungs following their inhalation.

## Introduction

Inhaled nanoparticles predominantly reach the alveolar space and settle on the alveolar epithelial cell (AEC) surface. For all major lung and some cardiovascular diseases, epidemiology shows a strong correlation with PM2.5 (aerosols with an aerodynamic diameter of up to 2.5 μm, consists mostly of the nanoparticles by number and toxicological significance), including ischemic heart disease, cerebrovascular disease, lung cancer, lower respiratory infections, and COPD^1^. This defines the need for a full understanding of these particles’ interactions with the body, including biokinetics and clearance mechanisms, the topic of the current investigation.

To study clearance, we chose amorphous silica nanoparticles (ASN) that are commonly found dust components in the ambient air and in the workplace^2^. In contrast to crystalline silica that causes or exacerbates lung diseases, such as silicosis, chronic bronchitis, chronic obstructive pulmonary disease (COPD), tuberculosis, or lung cancer^3,4^, amorphous silica has limited pathologic effects^5^, due to its rapid clearance. However, little is known about the mechanism of nanoparticle clearance after their inhalation and alveolar deposition. A kinetic study using silica-coated as well as other radiolabeled nanoparticles in rats showed a biphasic clearance from the lung within the first phase lasting minutes and the second phase characterized by roughly 1-day t_1/2_, leading to their complete removal^6^. Another study found that nanoparticles with noncationic surface charge rapidly translocate from the lung lumen to the mediastinal lymph nodes at a similar rate of clearance^7^. This speed and efficiency of removal of the ASN particles does not match the kinetics of alveolar macrophage clearance^8^, which takes weeks to years to complete for other nanoparticles^9,10^. Our recent study in mice instilled with labeled ASN, using intravital- and electron microscopy demonstrated that the first phase of the rapid ASN clearance is linked to their crossing of the AEC layer^11^. Silica- and other nanoparticles in the alveolar lumen form agglomerates, as is common for particles of this size in aqueous suspensions^12^. The agglomerates cross the epithelium in large, membrane-restricted compartments at a rate consistent with the reported biokinetics of clearance and thus much faster than any of the previously described transcytosis mechanisms for any known particles^13^. Once in the basal membrane- and interstitial region, the particles may either get cleared by the lymph^7^, or else directly cross the capillary wall into the bloodstream.

To determine the mechanisms involved in the process of uptake and the rapid transcytosis, we studied interactions of ASN with ultrastructural and molecular components of the primary and immortalized AEC pulsed with ASN. Results revealed a fundamentally novel, fast, and effective pathway for clearing ASN agglomerates, starting with endocytosis strongly enhanced by caveolae but was otherwise macropinocytotic. The ASN endosome was propelled along actin stress fibers tethered to a granule of the trans-Golgi network (TGN), rather than following microtubules. This novel TGN-linked transcellular transport, followed by basolateral exocytosis, was myosin II-dependent. In summary, our study shows the cell biology underlying an essential aspect of lung homeostasis.

## Results

The molecular mechanisms of particle transcytosis across the alveolar epithelium observed by us previously in situ in mice, was investigated in immortalized- and primary alveolar epithelial cell cultures (AEC) by microscopy as well as force spectroscopy (SCFS) in four steps, (a) adhesion to the apical membrane, (b) endocytosis, (c) intracellular transport, and (d) exocytosis at the basolateral membrane. The microscopy techniques employed are explained in Supplemental Fig. S1 and included electron microscopy (TEM) of thin sections (<100 nm), electron tomography (ET) of thicker sections (200 nm), confocal live cell imaging (LCI), structured illumination microscopy (SIM), and total internal reflection fluorescence microscopy (TIRF).

### Silica nanoparticles trigger active, strong adhesion in lung epithelial cells before internalization

**Figure 1:**
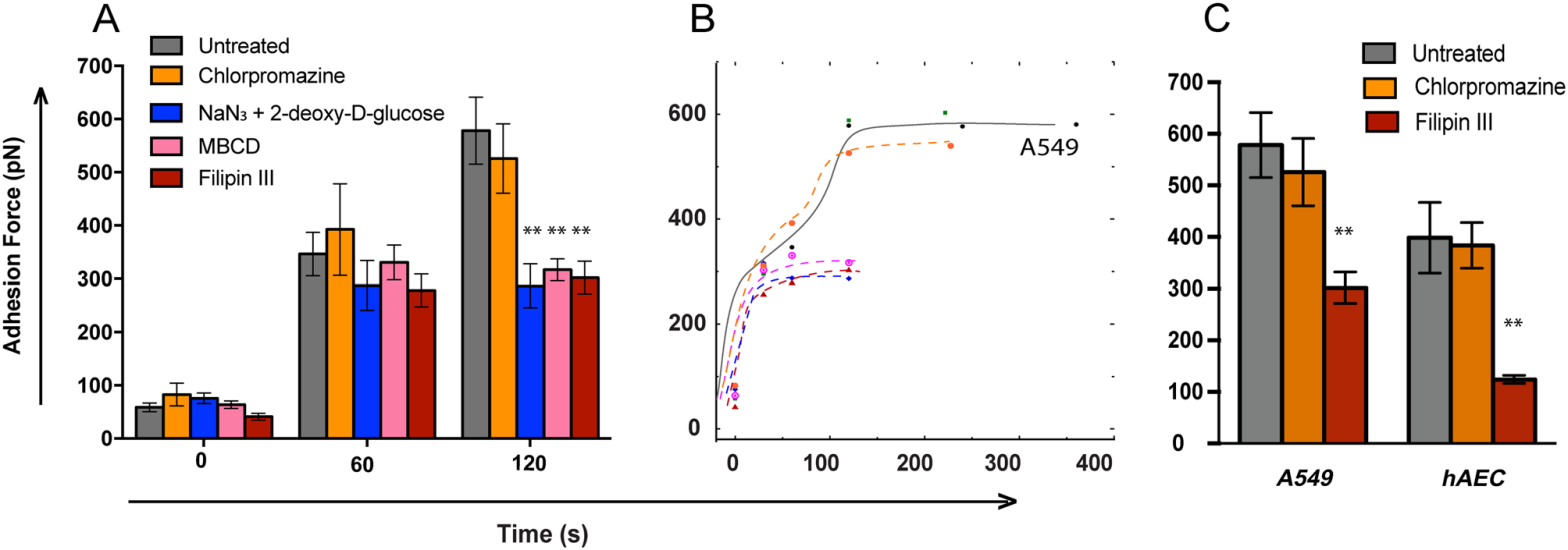
Firm adhesion of amorphous silica to alveolar epithelial cells (AEC) is energy and caveolin dependent. (**A**) Adhesion force between an amorphous silica probe tip and A549 cells was measured by force spectroscopy. Interfering with ATP production (NaN_3_ and 2-deoxy-D-glucose), lipid microdomains (LM, methyl-β-cyclodextrin MBCD) or caveolin (Filipin III) but not with clathrin-mediated endocytosis (chlorpromazine) diminished adhesion strength at 120 s, but not at the earlier time points. For each time point the data is collected from 10 different cells with an average 15 repeats on every cell with at least three independent experiments for each group (mean ± SEM; **p < 0.01 compared to matching untreated control). (**B**) Polynomial curve fitting of the dataset (from the initial 120 s and beyond) revealed biphasic process with the adhesion strength increasing between 0 and 30 s (not affected by the inhibitors) and 60–120 s (ATP-, LM- and caveolin-dependent). (**C**) Validation of the most important findings in primary human AEC (hAEC) at 120 s with no effect of chlorpromazine and significant suppression of the adherence strength by Filipin III (≥ 3 independent experiments with n = 10 different cells and an average 15 repeats on every cell).

Alveolar walls are constantly subjected to stretch and recoil forces during breathing. Thus, we hypothesized that the first step of ASN-AEC interaction must be firm ASN adhesion to the AEC apical membrane to prevent particles from becoming dislodged during initiation of the endocytosis. Adhesion of the amorphous silica (SiO2) to the apical surface of the AEC over time was measured using single-cell force spectroscopy (SCFS, the spectroscopy mode of an atomic force microscope, see schematic Supplemental Fig. S1A). Immortalized AEC lines, A549 and primary human alveolar epithelial cells (hAEC) were used to determine the binding force of SiO2 to their apical surface. The apex of an oxidized silicon probe with a 30-nm-diameter tip served as the equivalent to 30 nm ASN. The probe was brought into light contact (contact force <500 pN) with the apical surface of A549 and allowed to interact for a set contact time before retraction and measurement of the adhesion force. The adhesion force as a function of contact time (Fig. 1A,B) increased in a biphasic manner with an initial plateau at 30–60 s, followed by further increase before a maximum was reached at 120 s (Fig. 1B). Measurements in the presence of pharmacological blockers to specific adherence/endocytic pathway elements revealed that the immediate adhesion (up to 60 s) was not affected by any of the active binding cell membrane pathway blockers. However, the firm adhesion that occurred during the second phase of binding (60–120 s) could be abolished by the inhibitors. Energy dependence for the firm adhesion was demonstrated by the ATP blocker NaN_3_ plus 2-deoxy-d-glucose^14^. Its reliance on the presence of lipid rafts was demonstrated by abolishing the active adhesion component by methyl-β-cyclodextrin (MβCD)^15^, which disrupts cholesterol-rich membrane micro-domains. Finally, caveolae disrupter filipin III^16^ diminished the adhesion force to a similar degree as the other two inhibitors (p = 0.008), suggesting that caveolae harbor a receptor for the ASN or present an otherwise effective moiety for their firm adherence. In contrast, the inhibition of clathrin-dependent adhesion or phagocytosis^17^ with chlorpromazine (p = 0.69) did not affect the silica adhesion (p = 0.931) at 120 s (Fig. 1A,B). To establish translational significance, we verified our key observation in primary hAECs. Like in A549, in hAECs, filipin reduced the adhesion of oxidized silica by half (p < 0.01), whereas treatment by chlorpromazine, had no effect on adhesion (Fig. 1C). Thus, the firm adhesion of silica to the surface of the epithelial cell is an energy-dependent process that relies on the integrity of lipid rafts and caveolae formation but is clathrin-independent.

### ASN uptake by AEC combines mechanistic features of macropinocytosis with caveolar endocytosis

**Figure 2:**
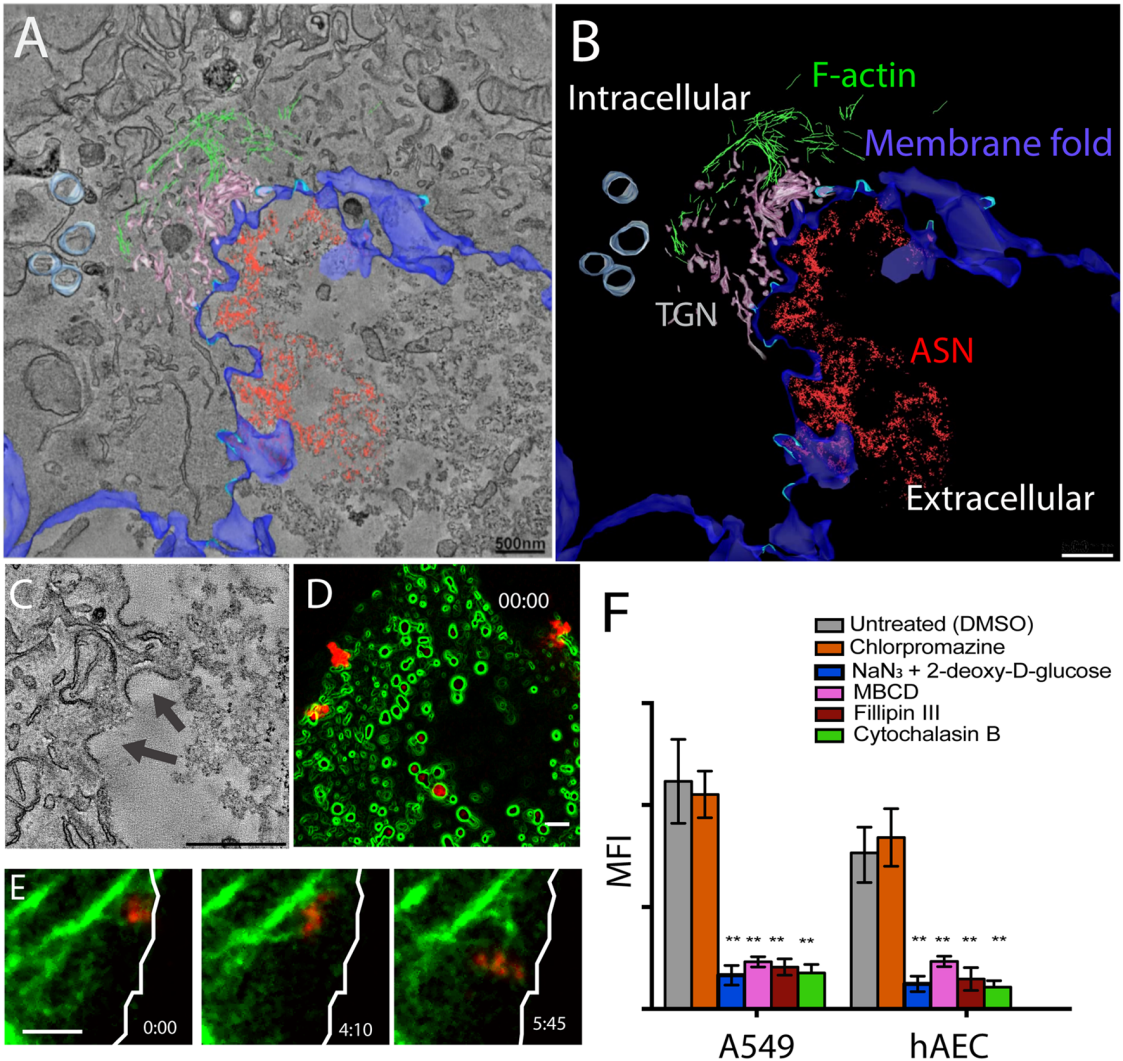
F-actin together with the factors driving firm adhesion of amorphous silica nanoparticles (ASN) to AEC, is responsible for ASN cellular uptake. (**A**) Electron tomogram captures the internalization of ASN by an A549 cell with the uptake compartment components marked as follows: F-actin (green); the plasma membrane (dark blue); ASN agglomerate (red); caveolae (cyan); the trans-Golgi network (TGN, beige); and mitochondria (light blue). (**B**) Ultracellular topography of the ASN uptake compartment components (structures annotated in panel A). (**C**) Identification of the protein-lined cup structures of 50 nm to 100 nm diameter, consistent with caveolae formation (black arrow heads) at the interface of ASN uptake. (**D**) mCherry-caveolin-1-transfected A549 cells form caveolae-lined invagination (pseudo-colored green) in a juxtaposition to the far-red-fluorescent ASN (red) during the uptake captured via confocal live cell imaging (LCI). (**E**) The involvement of actin during ASN internalization is revealed by LCI of eGFP-LifeAct-transfected A549. Note that actin bundles following or preceding the ASN agglomerates as they become internalized (cell boundary represented by white line). (**F**) Quantitation of ASN uptake in the absence and in the presence of endocytic inhibitors by A549 and primary hAECs. Note the ASN uptake dependence on all firm-cell-adhesion factors (per Fig. 1) as well as a significant uptake suppression by the actin inhibitor (Cytochalasin B). Mean ± SD, n = 4 **p < 0.01. Scale bar: (**A**–**C**) 500 nm, (**D**, **E**): 2 µm.

To visualize the subcellular components involved in ASN agglomerate internalization, we performed electron tomography (ET) of the uptake compartments. An electron tomogram (Fig. 2A and schematic Supplemental Fig. S1B, C) captures morphological details of the ASN uptake compartment at the apical surface of AEC at the time of its formation, prior to the cell membrane fusion. The ET was computed from 200 nm-thick tangential sections of the apical cell surface and the relevant structures annotated in color. The lining of the compartment (dark blue) contains cups (cyan) displaying the structure consisted with caveolae^18,19^ (Fig. 2A,B). A representative section of another tomogram (Fig. 2C) shows the caveolar cup structure in greater detail, with the regular pattern of caveolin apparent. We also observed bundles of F-actin fibers (green) organized at the intracellular portion of cell membranes invagination (Fig. 2A,B), while microtubules are not present in the area of active ASN uptake (also see Supplemental Fig. S2). Significantly, the endocytic cup membrane was always closely associated with the trans-Golgi-network (TGN) (Fig. 2A,B). Together, these morphological features are indicative of a novel uptake pathway. While the ultrastructure of the uptake compartment is consistent with macropinocytotic vesicle formation, the caveolar lining is not. As well, the direct association of the TGN with the endocytic cup is novel too.

To study the caveolar coating during endocytosis, we performed LCI in A549 cells transiently transfected with mCherry-caveolin-1. An optical section of the apical side of the cell (Fig. 2D and schematic Supplemental Fig. S1D) shows ASN agglomerates attached to a cup lined with caveolin-1 (Cav-1) during internalization. In summary, the ASN internalization structurally resembles macropinocytosis with an intriguing association of caveolae, TGN, and actin.

To understand the functional role of the association of actin to the particle compartment during uptake, we conducted confocal live-cell imaging with eGFP-LifeAct^20^ transiently transfected A549 cells. Time-lapse video demonstrated ASN agglomerates gradually submerging below the cell surface in conjunction with extending and retracting actin bundles (Fig. 2E, Supplemental Movie S1). Actin dissolution with cytochalasin B (CytB, Fig. 2F) abolished ASN uptake. Finally, the adhesion blockers identified by SCFS (NaN_3_ plus 2-deoxy-d-glucose , MβCD), filipin III also abolished ASN uptake (Fig. 2F), providing evidence that firm adhesion of ASN agglomerates to the epithelial membrane is an initial step of the uptake of the particles by the AEC.

**Figure 3:**
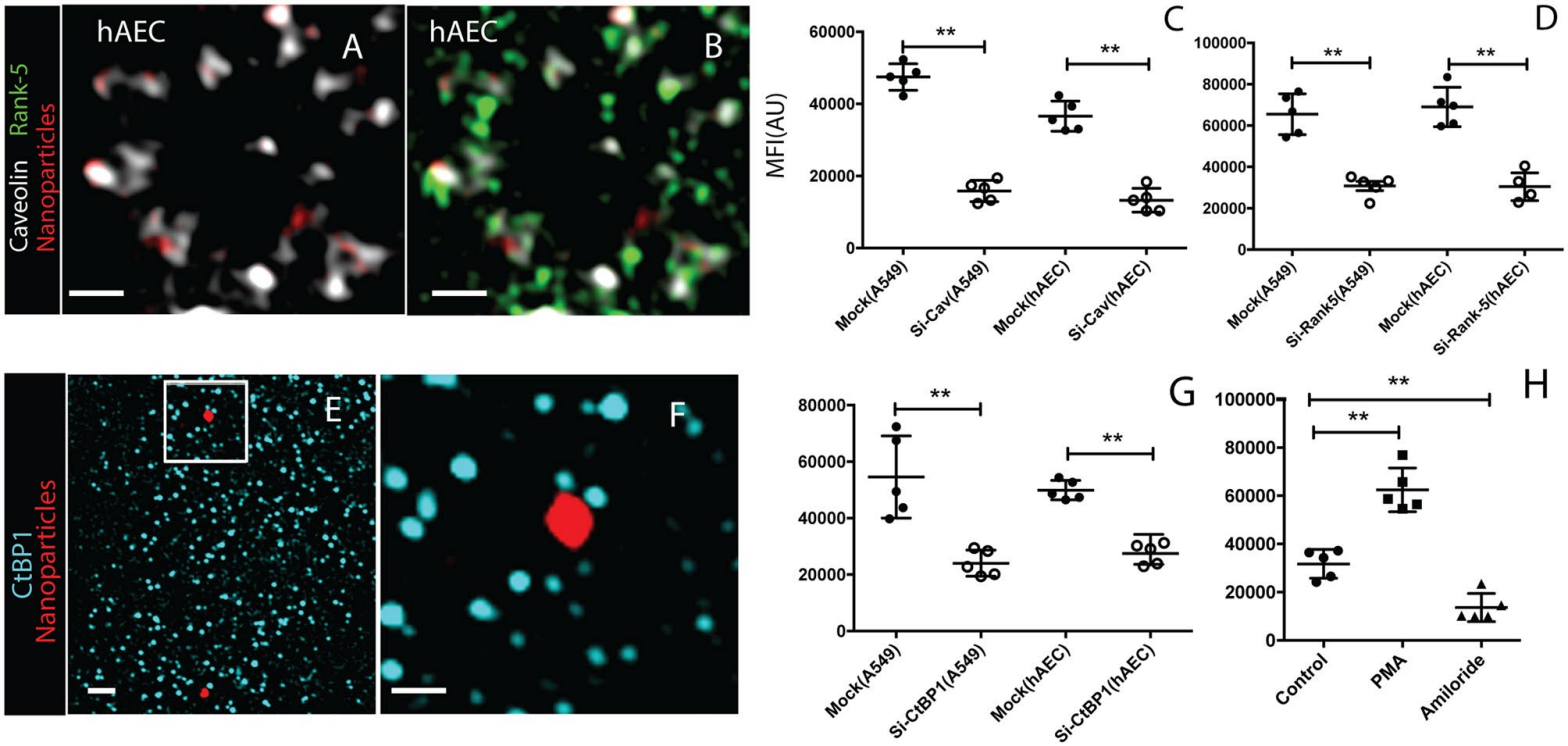
The ASN uptake by AEC combines mechanistic features of caveolar endocytosis with macropinocytosis. Structured Illumination Microscopy shows the proximity of elements for caveolin mediated endocytosis and macropinocytosis during ASN uptake. (**A**, **B**, **E**) Upon the hAEC-internalized ASN (red) are associating with both caveolin-1 (white, **A** and **B**) and macropinocytosis-markers Rank-5 (green, only **B**) and C-terminal Binding Protein 1 (CtBP1; blue **E)** (**F**) enlarged view of E. Knocking down by siRNA interference Cav-1 (**C**), Rank-5 (**D**) or CtBP1 (**G**), each reduced ASN uptake in A549 and hAEC by over 50% (mean from 100 cells ± SD, n = 5, **p < 0.01). (**H**) Macropinocytosis enhancer phorbol-12-myristate-13-acetate (PMA) nearly doubled ASN uptake while macropinocytosis-inhibitor amiloride reduced uptake by half in A549 cells. ASN uptake is expressed as MFI increase within a confocal slice of the cell that internalized ASN compared with untreated control. Charts show individual data points, mean from 100 cells ± SD, n = 5, **p < 0.01. Scale bars: (**A**, **B**, **E**): 1 µm, (**F**): 500 nm.

To delineate molecular detail of the caveolin-dependent but otherwise macropinocytic pathway, we immunostained hAECs with both Rabankyrin (Rank)-5, reported to be associated with macropinocytic vesicles^21^**,** and Cav-1 after exposing the cells to ASN. Super-resolution microscopy showed the association of both caveolin-1 and Rank-5 with the same uptake compartments in hAECs (Fig. 3A,B, A549 data in Supplemental Fig. S3 A-F). Furthermore, knocking down Rank-5 or Cav-1 by ~50%, assessed by qPCR, reduced the uptake by half in both A549 and hAECs (P = 0.007) (Fig. 3 C,D).

### Trafficking of ASN agglomerates occurs in caveolin-1 and Rank-5 coated vesicles, and is TGN associated

We next investigated how the uptake compartment closes and detaches from the apical surface of the cell to determine if the vesicle detachment pathway follows the canonical caveolar or the macropinocytic pathway. Dynamin that partakes in the scission of newly formed vesicles from the plasma membrane in clathrin‐dependent and caveolar endocytosis may be blocked by dynasore^22^. However, dynasore had no effect on the ASN uptake (p = 0.2031, Supplemental Fig. S4) at a concentration that inhibited ovalbumin uptake (control) by 77% (p = 0.002, Supplemental Fig. S4). In contrast, the closure of macropinosomes requires C-terminal-binding protein1 (CtBP1). We labeled CtBP1 in the hAECs and conducted super-resolution light microscopy. This showed CtBP1 in proximity but not overlapping with nanoparticle agglomerates (Fig. 3E,F). Consistent with macropinocytosis-driven ASN internalization, knocking down CtBP1 expression by siRNA interference reduced the uptake by more than 50% (Fig. 3G). Phorbol-12-myristate-13-acetate (PMA), an enhancer of macropinocytosis^23^, increased uptake 1.9 times (p = 0.003) and amiloride, a macropinocytosis inhibitor^24^, reduced the number of internalized agglomerates by half (p < 0.01) (Fig. 3H). Collectively, the involvement of Rank-5 and CtBP1 as well as the enhancing effect of PMA and inhibition by amiloride, implicate the ASN internalization mechanism to be macropinocytotic despite the unusual involvement of caveolin.

**Figure 4:**
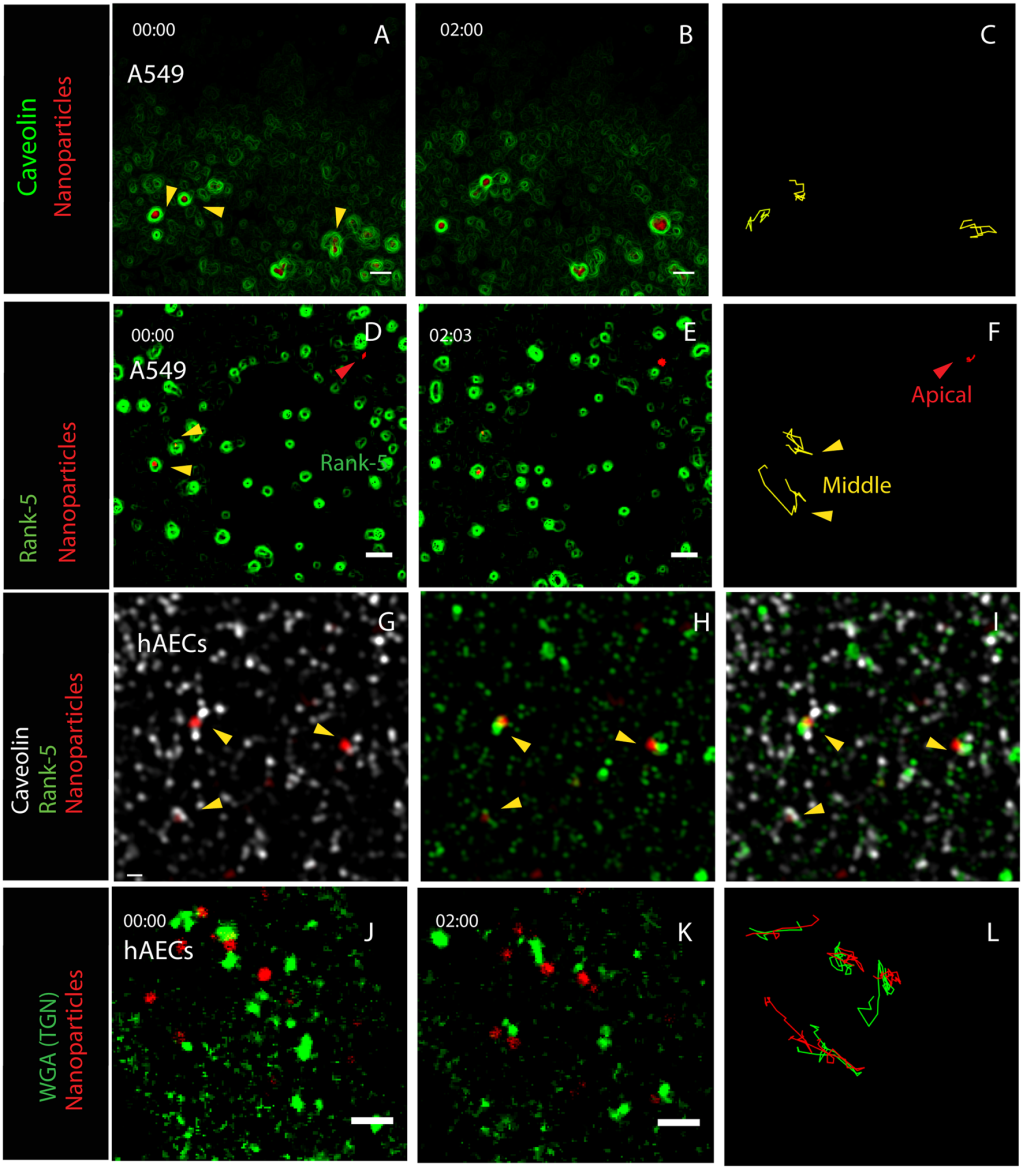
TGN is involved in intracellular trafficking of internalized ASN agglomerates within caveolin-1 and Rank-5 coated vesicles. (**A**–**C**) LCI images show ingested, far-red fluorescently-labeled ASN agglomerates (red) rapidly moving within the cell at 5.6 ± 1.88 μm/min. The transport occurs within a caveolin-1 lined vesicle as shown in mCherry-caveolin-1 (green pseudo-color)-transfected A549 cells. (**D**–**F**) Analogous images of A549, transfected with GFP-Rank-5 (green) show that at the cell surface the ASN agglomerates are not associated with Rank-5 (red arrow) and they do not move laterally; however, particles compartment acquires Rank-5 “lining” when the lateral movement commences at 5.9 ± 2.2 µm/min. (**G**–**I**) Structured illumination microscopy (SIM) of a fixed sample shows ASN associated with both caveolin-1 (white) and Rank-5 (green) in primary hAECs. (**J**–**L**) TGN granules (green) labeled with Alexa-488 wheat germ agglutinin (WGA) show TGN movement path coordinated but not overlapping with ASN agglomerates (red) in primary hAEC, and following somewhat different trajectories than TGN (green versus red lines) (**L**). Scale bars: (**A**–**F**,** J**–**L**): 2 μm, (**G**–**I**): 1 µm.

We investigated if both Rank-5 (macropinocytosis pathway) and caveolin-1 (caveolin endocytosis pathway) remain associated with the endocytic compartment during intracellular transport. LCI revealed that both caveolin-1 coated or rank-5 coated particle compartments were moving at the same rate of 5.5–6 µm/min (Fig. 4A–F). No significant differences in the velocity between the caveolin-1 coated vesicle and Rank-5 coated vesicle was observed (p = 0.6), providing indirect support of our hypothesis that both markers are likely part of the same compartment. Unlike caveolin-1, Rank-5, was not associated during the initial adhesion phase of the particles (Supplemental Movie S2), but recruited to the vesicle later, during the uptake and retained throughout the fast-moving transport phase (Fig. 4D–F, Supplemental Movie S3). Structured illumination microscopy (SIM) further showed that Rank-5 and Cav-1 label the same structures within the primary hAECs (Fig. 4G–I). SIM images showing similar localization of the endocytic elements relative to uptake compartment in A549 are presented in Supplemental Fig. S3A-F.

Another intriguing aspect of intracellular trafficking is the mode of locomotion for the ASN compartment. When we labeled the TGN with a wheat germ agglutinin-conjugated dye^25^, we found the association of the TGN with the particle compartment observed for the endocytic cup (Fig. 2A,B) maintained during the transcellular transport. In LCI each particle-containing vesicle traveled accompanied by a granule of the TGN tethered to it (Fig. 4J–L, Supplemental Movie S4). To determine whether such TGN association to endocytic vesicles would occur during classical macropinocytosis, AEC were pulsed with 90 kDa dextran agglomerates (a typical substrate for macropinocytosis used as a control here) and the resultant endocytic vesicles followed. No such association with dextran-loaded vesicles was observed (Supplemental Fig. S5), demonstrating its unique involvement hybrid mechanism of ASN uptake and their intracellular transport defined in this study.

### Basolateral plane exocytosis of ASN concludes the transport of the particles across the AEC

**Figure 5:**
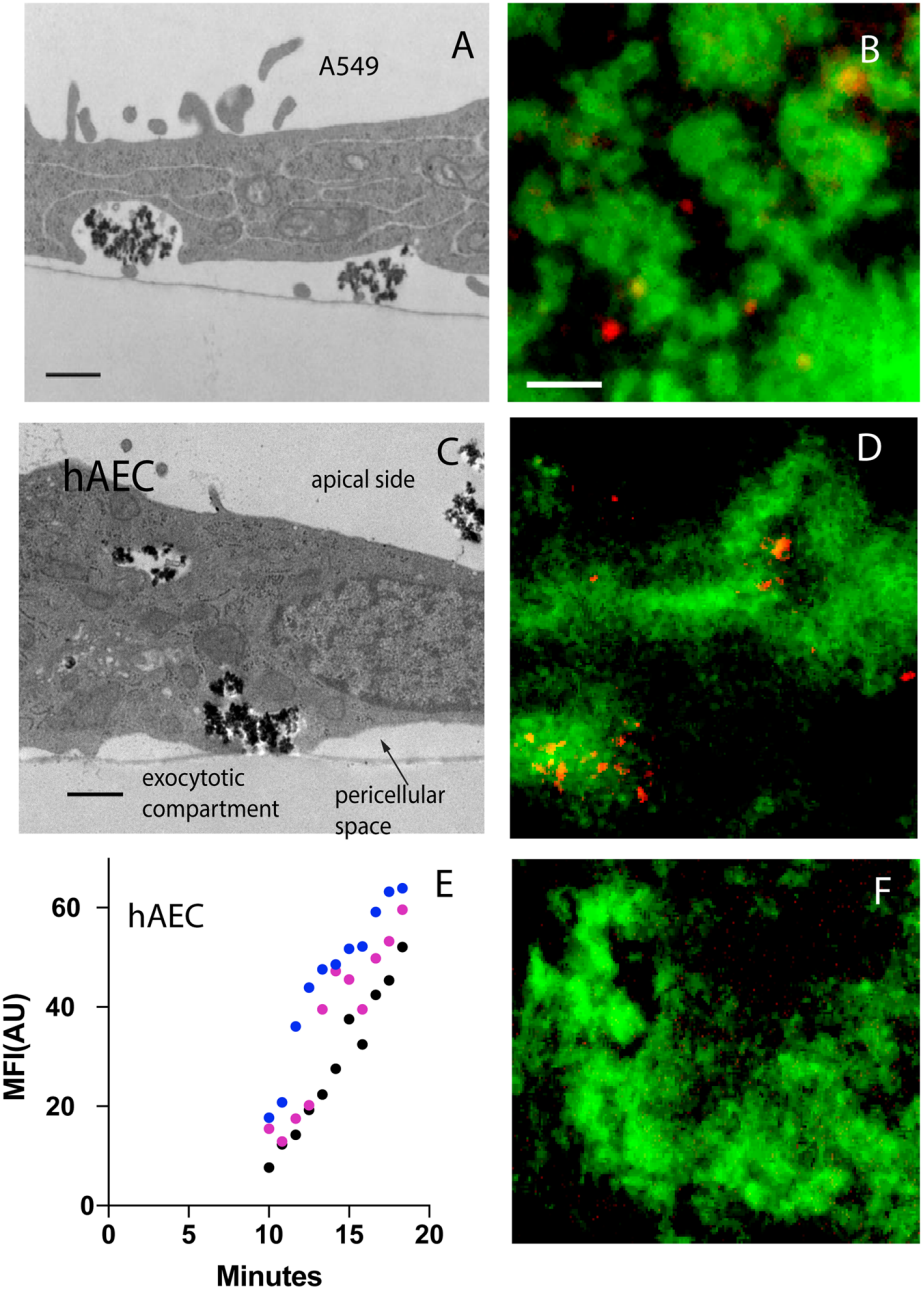
Transcellular transport of ASN internalized by immortalized (A549) and primary hAEC concludes with their exocytosis at the basolateral cell surface. (**A**) TEM visualization of ASN agglomerates at the basolateral surface of A549 cells attached to a culture dish and (**B**) their appearance in the TIRF microscopy field after exocytosis (red) from A549 (plasma membrane patches labeled with Alexa-488 WGA, green). TIRF was set to an illumination depth of < 100 nm. (**C**) Analogous ASN exocytosis images at the basolateral surface of primary hAEC cells in TEM and (**D**) in the TIRF field. (**E**) Analysis of three independent time lapse videos (as in **D**) demonstrate appearance of ASN in the TIRF plane starting at 10 min post their application to the apical plane of super confluent hAECs and the increase in far-red fluorescence intensity in consequence of labeled ASN exocytosis over time (n = 3). (**F**) This contrasts with analogous setup with PFA fixed cells (negative control) which do not allow for transfer of ASN to the basolateral plane within the equivalent time intervals. Scale bars: (**A**,** C**): 500 nm, (**B**,** D**,** F**): 5 µm.

Our previous study in mice showed that the rapid transcellular transport of ASN across the AEC barrier in mice is followed by exocytosis^11^. To examine this in greater details and to validate it in hAEC we examined ASN pulsed AEC with TEM. For these studies primary hAECs were cultured to obtain tight confluent monolayers and were pulsed with ASN on the apical surface to trigger their uptake. This was followed by vertical sectioning of cells has been conducted to observe ultrastructural details of exocytosis. The TEM shows ASN agglomerates found in the pericellular space between the coverslip and the cell in (Fig. 5A) supporting that ASN internalized via the apical AEC membrane were exocytose into pericellular space between at the basolateral cell aspect.

To visualize particle exocytosis in real-time, A549 cells and primary hAECs were studied using total internal reflection (TIRF) fluorescent microscopy. In TRIF (and TEM), we found the basolateral membrane of epithelial cells to adhere to coverslip in patches (adhesion patches) with the membrane detached from the coverslip in-between. Next, we set the illumination depth for TIRF to be extremely shallow (< 100 nm, by adjusting the angle of the excitation beam^26^) so as to illuminate only the coverslip-attached portions of WGA-labeled basolateral plasma membrane^27^ and leaving the non-adhered “pockets” of cell membrane and the cell interior in the dark^28–30^. Time laps movies were acquired to establish the kinetics of the ASN entering the pockets between the basolateral membrane and the coverslip (pericellular space). For A549 recordings (Fig. 5B) showed gradual appearance of labeled ASN at the basolateral space starting at 20 min after the apical ASN application. For primary hAEC (Fig. 5C,D), ASN fluorescence in pericellular space between WGA patches could be detected as early as 10 min after the addition of nanoparticles (Fig. 5D). The numbers of particles increased over time (Supplemental Movie S5). Quantification of the ASN fluorescence showed increasing fluorescence intensity occurring in a linear fashion between 10 and 20 min (Fig. 5E), indicating accumulation of exocytosed particles trapped in the pericellular space below the AEC.

To ensure that the particles that appeared in the pericelluar space originated from cells, confluent hAECs were incubated for 1 h with ASN before particles were removed and confocal images were taken either from a mid-plane, or a maximum intensity projection of the entire cell volume was conducted. A significant reduction in mean fluorescence intensity inside the cells was shown after the apical exposure to ASN was removed (Supplemental Fig. S6 A-D) providing evidence that internalized ASN cleared from the AEC cell interior within the time course consistent with their exocytosis. Removing, lysing the cells, and measuring the total fluorescence gave a similar result (Supplemental Fig. S6 E). As a final control, fixing hAEC monolayers with paraformaldehyde- prior to ASN exposure, blocked ASN appearance at the basolateral aspect (Fig. 5F). Together, these findings demonstrate that the transcellular ASN transport across the AEC monolayer is active, efficient and concludes within a matter of minutes with the exocytosis at the basolateral membrane.

**Figure 6:**
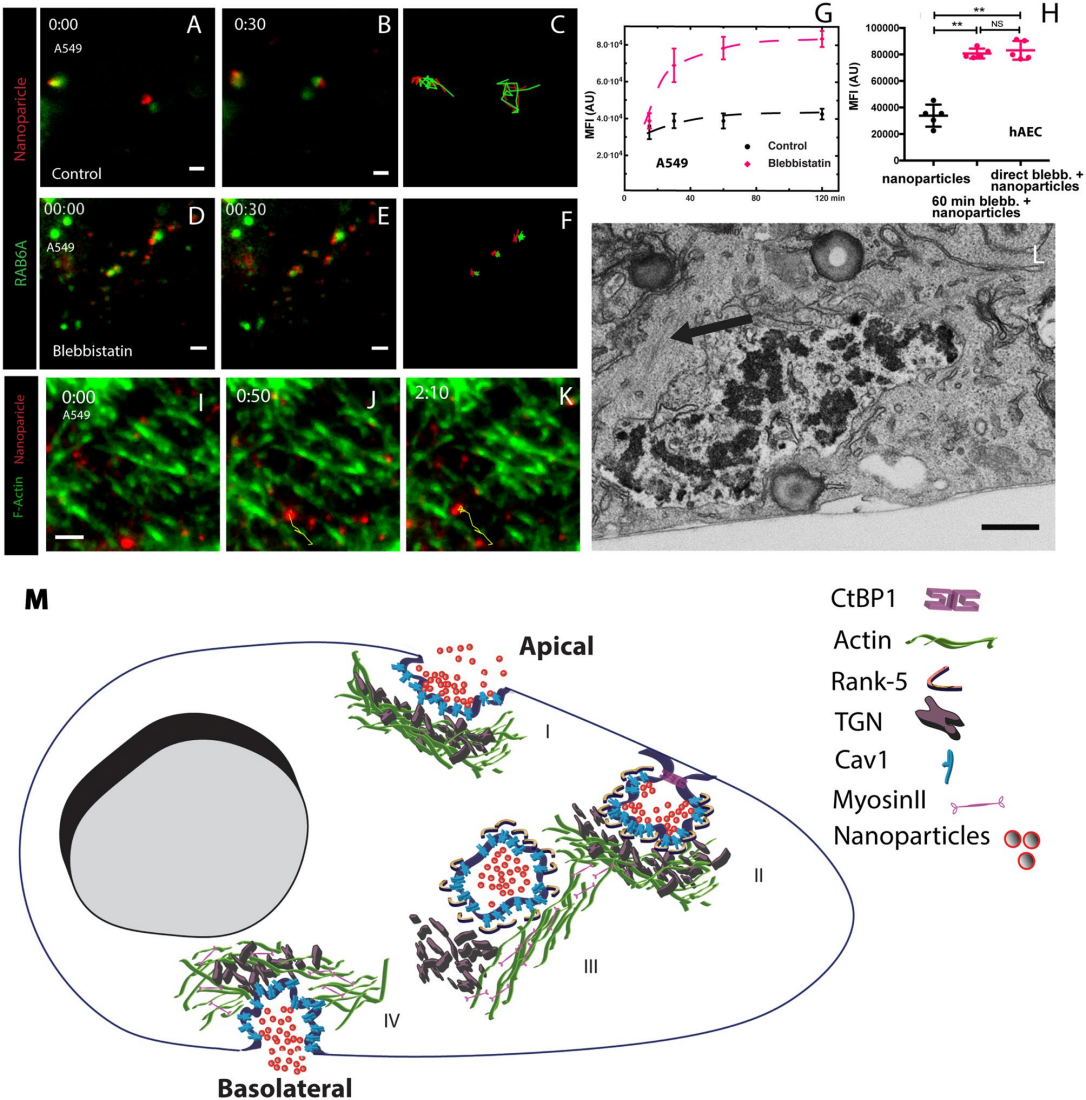
Actin, myosin II, and Rab6A are engaged in the ASN-containing vesicle transport for exocytosis. TIRF microscopy at the basolateral side (illumination depth: < 250 nm). (**A–C**) GFP-Rab6A (green) marks the TGN and exocytotic vesicles and highlights the correlated movement of the TGN granule and with associated particle agglomerates (red). (**D**–**F**) Blebbistatin, a myosin II inhibitor, blocks the movement of the ASN compartments and the associated TGN. (**G**) The particles accumulate inside the cells in presence of blebbistatin. Data represent mean ± SD, n = 3. (**H**) Blebbistatin preincubation does not block the ASN uptake but their transport and exocytosis, since accumulated inside the pre-treated hAECs (Data are mean ± SD, n = 3 and **p < 0.01). (**I**–**K**) Labeled ASN-containing compartments (red) move along actin stress fibers (green) near the basolateral surface of A549 cells. The yellow track indicates the trajectory of a large agglomerate at the basolateral side. The agglomerates move along actin fibers at 4 microns during 50 s (Supplemental Movie S8). (**L**) Electron micrograph showing a particle ASN compartment adjacent to an F-actin bundle (black arrow) at the basolateral edge of the A549 cells. (**M**) A model of nanoparticle agglomerate transcytosis in lung epithelial cells. Formation of the uptake compart. Particles (red) adhere to the plasma membrane (dark blue) in a caveolin-dependent (cyan) way before the compartment forms (**I**). During internalization by actin (green) remodeling, the compartment remains associated with caveolin-1 and is cleaved off the plasma membrane by CtBP1 (magenta). At this point, Rank-5 (brown) is recruited to the compartment (**II**). The movement of the agglomerate compartment depends on Myosin II (pink). The compartment appears to be propelled by a trans-Golgi network (TGN) granule that moves along actin stress fibers. The TGN granule and the particle compartment are tethered to each other with a distance of up to 1 µm (**III**). Exocytosis of the particles after Rab6A is recruited to the TGN granule associated with the agglomerate-compartment. Rank-5 is removed at this point (**IV**). We have not directly observed the association of the TGN with its immersed actin network, myosin II and the actin stress-fiber and how this leads to the directed movement, nor is the nature of the tether known. The depiction is therefore based on the observation of function and is not literal. Scale bar (**A–C**) 2 μm, (**D**–**F**) 2 µm, (**L**) 200 nm.

Next, to determine if ASN cargo vesicles are targeted for exocytosis in AEC, we transiently transfected A549 cells with GFP-Rab6A. Rab6A is a known factor targeting carrier vesicles for exocytosis and a regulator of the cellular transport of these vesicles^31,32^. We imaged the basolateral portion of these cells using a TIRF incidence angle set to illuminate < 250 nm after pulsing the apical side of the cells with ASN. We found that the ASN cargo vesicles had an association with Rab6A, however, Rab6A seemed not to be directly targeting the ASN vesicles, but rather targeted TGN granules paired with the ASN compartment (Fig. 6A–C, Supplemental Movie S6). This unique spatial relationship supports that TGN provides locomotion and docking of the ASN vesicles at the exocytotic plane but does not fuse with the membrane of the ASN cargo compartments. This notion was further supported by experimental dissolution of Golgi with golgicide A, which resulted in an accumulation of the particles within the cells (Supplemental Fig. S7). This experiment also showed that the TGN was not essential for ASN uptake but required for shuttling ASN vesicles to the basolateral for particle export.

The transport of such a large Rab6A-tagged TGN-ASN-vesicle complex and its docking to the cell membrane would require an active involvement of the cytoskeletal motility apparatus, such as actin-myosin interactions. Consistent with this hypothesis, the treatment of the cells with the myosin II inhibitor blebbistatin inhibited joint locomotion of the complex, while preserving the relationship of the TGN-particle compartment pair (Fig. 6D–F, Supplemental Movie S7). Treatment with blebbistatin also resulted in a significant accumulation of ASN agglomerates within A549 and primary hAECs (Fig. 6G,H), showing that the ASN uptake was independent of myosin II in contrast to ASN export/exocytosis. Blocking myosin II with blebbistatin in hAECs 1h prior to nanoparticle addition did not affect particle uptake (Fig. 6H).

To evaluate the actin-myosin interactions, we used again TIRF microscopy illuminating < 250 nm of the basolateral side of GFP-life-act^20^ transfected A549 cells. The trajectory of the labeled ASN followed the actin stress fibers in straight lines. Occasionally, the compartments transitioned from one bundle to another evidenced by sharp change of direction (Fig. 6I–K, Supplemental Movie S8). Hence, stress fibers provide the scaffold along which the ASN vesicles reach the basolateral membrane for exocytosis. This is also apparent in TEM of thin sections (Fig. 6L).

In conclusion, TGN-granule is targeted by Rab6A for exocytosis and propelled by myosin II along stress fibers transporting the attached ASN-cargo vesicles to their basolateral exocytosis docking sites.

## Discussion

Our previous study identified a process of rapid nanoparticle transcytosis across the alveolar epithelium following their inhalation by mice^11^. This process initially supported by biokinetic studies and captured intravital microscopy in mice lungs^11^ applies to agglomerates representing the natural state of most nanoparticles in aqueous suspension^12^. In the present study, we dissected molecular mechanisms orchestrating this rapid and effective removal of nanoparticles from the alveolar lumen. This mechanism encompasses endocytosis that is strongly enhanced by caveolae but was otherwise macropinocytotic followed by the ASN endosome transport propelled along actin stress fibers tethered to a granule of the trans-Golgi network (TGN), rather than following microtubules.

We employed several microscopy approaches to establish a sequence of events summarized in Fig. 6M I–IV. The initial step is the arrest of particle agglomerates at the location of contact resulting in a strong initial binding that rapidly increases in the first 60 s after contact. This observation is in keeping with intravital microscopy findings where particle agglomerates deposited at the alveolar epithelial surface become immobilized with no apparent lateral movement on the epithelium^11^. Upon initial contact of an agglomerate with a cell, individual particles protruding from the agglomerate perimeter will make a multi-point contact within a region of the plasma membrane. Additionally, recent studies by Francia et al. have shown that BAR (Bin/amphiphysin/Rvs)-domain proteins, sensing and inducing membrane curvatures, play a significant role in the initial cellular binding process of silica nanoparticles as well^33^.

Our next fundamental finding, the epithelial cells employ a novel mechanism of nanoparticle uptake that represents a hybrid between caveolar- and macro pinocytotic mechanisms. Our data provide evidence that each contact point with the agglomerate triggers the initiation of a caveolar endocytosis. Thus, disrupting caveolae or downregulating caveolin-1 both strongly reduce both the firm adhesion and uptake. We propose that as the submersion of individual caveolin-anchored contact points progresses, the formation of a larger membrane contact surface sterically diverts the conclusion of the canonical caveolae-dependent endocytosis, reserved for particle diameters of < 70 nm^34^ and the internalization continues as macropinocytosis. Consistent with this view, downregulation of dynamin responsible for scission of caveosomes from the plasma membrane has no effect on the uptake studied here, whereas adapter family proteins involved in macropinocytosis—Rank-5, and Ankyrin^21^, as well as CtBP1 that takes part in the scission of macropinosomes from the plasma membrane, are all required^35^.

Once inside the cell, trafficking of the particles across the cell shows a novel role for the trans Golgi network (TGN). During internalization, while still open to the extracellular space, the endosome firmly associates with a granule of the TGN of comparable size to the particle compartment, an association that remains in place for the remainder of the passage of the particle cargo. After the endosome is detached from the plasma membrane, the two compartments are propelled across the cell tethered to each other (Fig. 4J–L). Transport is along actin stress fibers (Figs. 2E, 6I–K) and Myosin II-dependent rather than along microtubules. This TGN-cargo train has not been previously described but may occur in the cell on other occasions as well. Consistent with such mechanism, Rab6 family proteins and myosin II were shown to regulate the fission of Rab6-positive carriers^36^, and Rab6A promotes docking of endosome-derived vesicles to the plasma membrane^37^, including for exocytosis^38^. Interestingly, in our study the TGN granule, but not the cargo compartment, stained with WGA and Rab6A, suggesting that the TGN promotes the locomotion across the cell and then docks the cargo to the basolateral membrane for exocytosis, though does not share its membrane with the cargo compartment.

Our study, in addition to showing the cell biology underlying an essential aspect of lung homeostasis by clearing this region, also offers a new aspect for the understanding the devastating health effects of particulate air pollution^39^. Nanoparticles are constantly inhaled into the lungs^2^, hence rapid clearance across the epithelium may render otherwise toxic nanoparticle relatively benign. On the other hand, particles that are unable to access this pathway may lead to an extended, possibly life-long, exposure of the vast surface area of the alveolar plane^10^. Removal via mucociliary escalator^8^ is slow for the alveolar region, owing to the scarcity of alveolar macrophages that need to ingest the particles first in the absence of ciliated cells below the bronchiole. Furthermore, the described process is fundamentally different in macrophages, where a co-localization with the endosomal-lysosomal pathway is present after endocytosis of plain silica nanoparticles^40^.

While silica nanoparticles of around 50 nm have been reported to be more efficiently taken up compared to smaller or larger particles^41^ , different sizes and surface charges can alter the uptake mechanism^42,43^ and should be investigated further. In addition, the cellular uptake mechanism is heavily dependent on the cell type^44^, serum proteins coating the nanoparticles are also crucial for the uptake behavior^44,45^. Whereas the LDL-receptor has been described to play a significant role in silica nanoparticle uptake^46^, it is not linked to the usual clathrin-mediated uptake mechanism^33^.

It has to be noted that for our *in vitro* studies we used A549 cells, representing more type II pulmonary epithelial cells, and primary hAECs, which are a mixture of type I and II cells. Considering that most surface area of the alveoli, and therefore contact area to particles, are built up by type I cells, we don’t know if and how these cell types play different roles in nanoparticles transcytosis.

A further limitation of our study is that we predominantly used microscopy techniques to elucidate the transcytosis pathway of our particles, which primarily results in qualitative conclusions. Even our quantitative image analyses remain on a limited number of cells analyzed, which have to be verified with orthogonal techniques, such as radioactive labeling, to validate a quantitative statement.

Nevertheless, our presented data only focuses on the cellular mechanism, but in combination with other quantitative studies^6,11^, this pathway is likely to play a substantial role in silica nanoparticle lung clearance. Hence, our described pathway contributes to a mechanistic understanding of the initial phase of a bi-phasic clearance of silica-coated and radiolabeled nanoparticles published by Konduru et al.^6^.

In conclusion, the current study describes a novel transcytosis mechanism, which we propose is a key aspect of lung homeostasis keeping the lung free of nanoparticles. It will be important to determine whether and to what degree the disruption of this pulmonary clearance mechanism contributes the development pulmonary diseases.

## Materials and methods

### Nanoparticles

Non-fluorescent amorphous silica nanoparticles with an indicated Ø of 10–20 nm (BET) were purchased from Sigma-Aldrich (Ontario, Canada) and have a ζ-potential of − 24.4 ± 1.0 mV in 1.5 mM NaCl at 25 °C (Zetasizer Ultra Red, Malvern Panalytical Ltd, Malvern, United Kingdom). Red-fluorescent plain amorphous silica nanoparticles with a nominal Ø 50 nm (excitation/emission: 569/585 nm) were purchased from Kisker biotech (Steinfurt, Germany) that have been reported with hydrodynamic size (z-average), PDI, and ζ-potential of 47 nm, 0.07, and − 40 mV, respectively^44^. The custom-made far-red-fluorescent particles (Kisker biotech, excitation/emission: 759/779 nm) had a hydrodynamic size (z-average), PDI, and ζ-potential in 1.5 mM NaCl at 25 °C of 67.2 ± 0.9 nm, 0.27 ± 0.01, and − 40.7 ± 2.1 mV, respectively (Zetasizer Ultra Red) and were described in our previous publication^11^. The particles were stored as a stable colloid at 25 mg/ml.

### Cell culture

A549 was obtained from ATCC, Virginia, USA, and maintained in F12 (Gibco, USA) supplemented with 10% of fetal bovine serum (Gibco, USA), 2 mM L-Glutamine, and 10 mM Hepes (Life Technologies, Canada). Commercially obtained Human Primary Alveolar Epithelial Cells (hAECs) (Cell Biologics, USA) were grown in HuMEC Basal Stirferum Free medium (ThermoFisher Scientific, USA) supplemented with HuMEC supplement Kit (ThermoFisher Scientific, USA).

### Single cell force spectroscopy

A Nanowizard-II AFM (JPK instruments, Germany) was used to measure the adhesion force between nanoparticles and cells. To mimic silica nanoparticles, silicon AFM tips with a diameter of 30 nm (HYDRA2R-100NG-50, APP NANO, USA) were used as it oxidizes at the surface to produce a silica-interface. As a control, silicon tips with an additional 1 nm-layer of amorphous silica coating using a JohnsenUltravac medium throw e-beam evaporation system were used (coating performed at the National Institute of Nanotechnology Edmonton, Canada). Adhesion forces for the uncoated and coated tips to cells were not statistically different (data not shown). For experiments with micro-amorphous-silica particles, individual particles were glued to tipless cantilevers using epoxy glue.

For adhesion measurements, the tips were approached (approach/retract rate: 2 μm/s; z-range: 10 μm) to the cell until a pre-set load of 0.5 nN or less was reached. After contact, the cell and tip were separated and the adhesion force was measured and plotted as a function of the contact time (0–120 s). The effect of contact time on the adhesion force was obtained for at least 10 different cells at different experimental conditions. The adhesion force was measured in F12-K medium supplemented with penicillin (100 units/ml), streptomycin (100 μg/ml), fungizone (0.25 μg/ml) and 10% FBS (in the presence of inhibitors for the inhibition studies) at 37 °C and 5% CO_2_. Inhibitors, including chlorpromazine (10 µg/ml), cytochalasin B (1–5 µg/ml), filipin III (1 µg/ml), methyl-β-cyclodextrin (MβCD, 2–10 mM), sodium azide (NaN_3_, 10 mM), and 2-deoxy-d-glucose (5 mM) were purchased from Sigma-Aldrich (Ontario, Canada). For the inhibition study, all the inhibitors were added 30 min prior to the experiment with fresh culture media.

### Electron microscopy and tomography

Cells were exposed to particles for 2 h and then fixed with 1.6% formaldehyde and 2.5% glutaraldehyde in 0.1 M sodium cacodylate buffer, pH 7.3 for 1 h, then post-fixed with 1% osmium tetroxide for 1 h in 0.1 M sodium cacodylate. Cells were dehydrated through graded ethanol and then embedded in Epon. After polymerization 70 nm thin vertical sections were cut from a representative area with cells with a diamond knife on an ultramicrotome (Ultracut E, Reichert-Jung, Vienna, Austria). Sections were mounted on electron microscopy grids and were stained with aqueous uranyl acetate and Reynolds’s lead citrate and observed under a Hitachi H-7650 TEM (Hitachi) at 80 kV with an AMT16000 digital camera (Advanced Microscopy Techniques, USA).

Electron tomography was performed on a Tecnai F20 G2 FEG-TEM (FEI, USA) with a Fischione 2040 Dual-Axis Tomography Holder (Fischione Instruments, USA). Cells were embedded as described above and 200 nm thick sections were cut parallel to the sample plane and mounted on a Formvar-coated TEM slot grid (1 × 2 mm). Colloidal Au particles of either 15 nm or 20 nm diameter were placed on both sides of the Formvar film to serve as fiducial markers. Finally, a thin carbon coating was applied to both sides of the grid to reduce electrical charging in the microscope. TEM tomography was done by taking digital images on a GatanUltrascan 4000 digital camera (Gatan, USA) and the microscope controlled using the open source software SerialEM (The Boulder Laboratory For 3-D Electron Microscopy of Cells, USA) as described^47^. The tomographic reconstruction was done by weighted back-projection with the IMOD open source software (The Boulder Laboratory for 3-D Electron Microscopy of Cells, USA), also used for visualization and analysis.

### Live cell imaging and transient transfection

Live cell imaging was done in a Zeiss Elyra, using an LSM-780 scanner. TIRF imaging was performed using an EMCCD camera (Andor). To visualize the plasma membrane and the TGN, cells were exposed to 10 µg/ml Wheat Germ Agglutinin (WGA) Alexa-488 conjugates (Life Technologies). eGFP-Lifeact (ibidi GmbH, Gräfelfing, Germany) transfected A549 were used to look at F-actin fibers with Ø 50 nm red fluorescent nanoparticles. mCherry-caveolin-1 (Gift from Ari Helenius’s lab, through Addgene, USA) transfected A549 cells, together with far-red Ø 50 nm fluorescent nanoparticles were used to establish the role of caveolin in particle trafficking. Rank-5 nanoparticle interaction was visualized by eGFP-Rank5 (Gift from Steve Caplan, University of Nebraska Medical Center, USA), transfected A549 cells, and Ø 50 nm red fluorescent silica nanoparticles. Interaction of Rab6A with nanoparticles at the TIRF plane was examined with eGFP-Rab6A (Gift from Anna Akmanova, Utrecht University, Netherlands) and transfected A549 cells. For all experiments, 2–2.5 µg of 50 nm nanoparticles per 35 mm µ-dish (ibidi) were used (growth area 3.5 cm^2^). Transfection was carried out by Lipofectamine 2000 (Invitrogen, USA) using the protocol supplied by the vendor.

### Structured illumination super-resolution microscopy (SIM)

Rabbit polyclonal anti-ANKFY1 (Rank-5) IgG (Abcam, USA), mouse monoclonal anti-Caveolin-1 IgG (Abcam, USA), rabbit polyclonal anti-CtBP1 IgG, and rabbit polyclonal anti-TGN IgG (trans-Golgi network) were used for immunofluorescence. Cells grown on cover slips were fixed using 2.5% paraformaldehyde in PBS for 10 minutes and then permeabilized with 0.5% Triton X-100 in PBS for 10 min. Coverslips were incubated with 1 µg/ml of primary antibody for 1h at 37 °C. Appropriate secondary antibodies including Alexa-488 F(ab’)2 fragments of Goat anti-mouse IgG, Alexa-488 Goat anti-rabbit IgG (Life Technologies, USA), Cy5.5 Goat anti-mouse IgG, or Cy5.5 Goat anti-rabbit IgG were used to visualize the structure of interest. All the secondary antibodies were checked for background staining, without primary antibodies, before performing the actual experiment. Secondary antibody was incubated for 1h followed by 30 min wash in PBS. For all immunofluorescence experiments 50 nm red fluorescent labeled nanoparticles were used. To visualize F-actin, phalloidin 647 (ThermoFisher Scientific, USA) was used. Coverslips were mounted with Prolong Gold (containing DAPI) before visualization. Imaging of processed A549 cells was done by inverted super-resolution microscope ELYRA PS.1 equipped with 63x 1.4NA objective (Zeiss, Germany). For structured illumination microscopy (SIM) each channel was acquired using five rotations, five phases, and 0.1 µm steps in z-axis. Raw images were reconstructed using Zeiss ZEN 2012 software (Zeiss, Germany).

### Total internal reflection fluorescence

For TIRF imaging the sample was illuminated below the critical angle and set to an evanescent field illuminating of either < 100 nm or < 250 nm above the glass/sample interface by adjusting the angle of incidence^26,48^. Excitation was performed with a nominal laser power of 100 mW and emission signals in the green and red channel were sequentially acquired with a 495–575 nm and 570–650 nm bandpass filter, respectively. Images were acquired using an AndorIXon 897 EMCCD camera (Belfast, UK) with an exposure of 100 ms for each color channel. Images were acquired by either a Zeiss ELYRA (Zeiss, Germany) with a 100 X TIRF objective or by a Diskovery Flex Microscope (Quorum) and a 60X TIRF objective. For negative control experiment, confluent monolayers of hAEC were fixed by 5 % PFA for 10 minutes before the TIRF experiment.

### Uptake and inhibition studies

For quantitative uptake experiments, 2–2.5 µg of red or far-red 50 nm nanoparticles were added to a confluent monolayer of A549 cells or hAECs in a 35 mm µ-dish (ibidi) for 1h and then washed to remove unbound nanoparticles. Samples were then fixed with 2.5% PFA for 10 min and visualized by confocal microscopy. Stacks were recorded 0.3 µm apart from apical side to basolateral side. A representative plane near to the middle of the stack was chosen for mean fluorescence intensity (MFI) calculation and to ensure the particle was within the cell. By using imageJ MFI was calculated from 5 arbitrary areas within every cell and a minimum of 20 cells were counted to obtain mean fluorescence intensity/unit area (MFI/µm^2^). A minimum of four experiments were performed. Alternatively, we have also measured total fluorescence from a maximum intensity projection of the all the optical sections. Both measurement strategies showed a similar result. Particle uptake for cells at the middle of the stack was quantified using the imageJ analyze particle plug-in. Additionally, uptake inhibitors or enhancers were used including chlorpromazine (10 µg/ml), cytochalasin B (1–5 µg/ml), filipin III (1 µg/ml), methyl-β-cyclodextrin (MβCD, 2–10 mM), sodium azide (NaN_3_, 10 mM), and 2-deoxy-D-glucose (5 mM), dynasore (1 µg/ml), phorbol-12-myristate-13-acetate (PMA) (0.5 µg/ml), amiloride (1 µg/ml), blebbistatin (2 µg/ml) (Sigma, USA), golgicide A (1 µg/ml) (Sigma, USA). For the inhibition study, all the inhibitors were added 30 min prior to the experiment with fresh culture media.

A set of siRNA were also used for uptake study. Caveolin-1 siRNA r(CCACUCAGCAACUGAAUGA-dTT) was synthesized at the University of Calgary, Canada. Rabankyrin-5 siRNA (Trisilencer-27 siRNA) was obtained from Origene RNAi, USA), and CtBP1 siRNA human, was obtained from Santa Cruz Biotechnology, USA). All the procedures were performed as described according to standard protocol of Transfecting Stealth RNA and siRNA using Lipofectamine 2000 (Invitrogen Life Technology, USA). Thus, 1 day before transfection A549 were plated at 3 × 10^4^ cells in 400 µl of growth medium without antibiotics per well. Cells reached 60% confluence by the transfection time. For each transfection sample siRNA-Lipofectamine 2000 complexes were prepared by mixing 50 pmol of siRNA in 50 µl of Opti-MEM Reduced Serum Medium plus 1 µl of Lipofectamine 2000. After transfection medium was replaced after 4–6 h. Knock down was assayed between 24 and 48 h after. Uptake experiments described above were performed after 48 h.

### Statistical analysis

Statistical analysis was carried out using GraphPad Prims 6 (GraphPad Software, San Diego, CA. Multiple comparison was performed with a one-way ANOVA followed by a Tukey post hoc test. Statistical significance on plots are indicated as follows: *P < 0.05, **P < 0.01, ***P < 0.001.

## Supplementary Information

Supplementary Video 1.

Supplementary Video 2.

Supplementary Video 3.

Supplementary Video 4.

Supplementary Video 5.

Supplementary Video 6.

Supplementary Video 7.

Supplementary Video 8.

Supplementary Figures.

## Data Availability

All data needed to evaluate the conclusions in the paper are present in the paper and/or the Supplementary Materials. Additional data related to this paper may be requested from the authors.
