# Supplementary material for: Caveolin-initiated macropinocytosis is required for efficient silica nanoparticles’ transcytosis across the alveolar epithelial barrier
Source: Sci Rep. 2022 Jun 8;12:9474. doi: 10.1038/s41598-022-13388-7 (PMC9178038; doi:10.1038/s41598-022-13388-7)
Supplement: Supplementary file 9 — Supplementary Figures. [file 41598_2022_13388_MOESM9_ESM.docx]

**Caveolin initiated macropinocytosis is required for efficient silica nanoparticles’ transcytosis across the alveolar epithelial barrier**

**Supplementary Material**


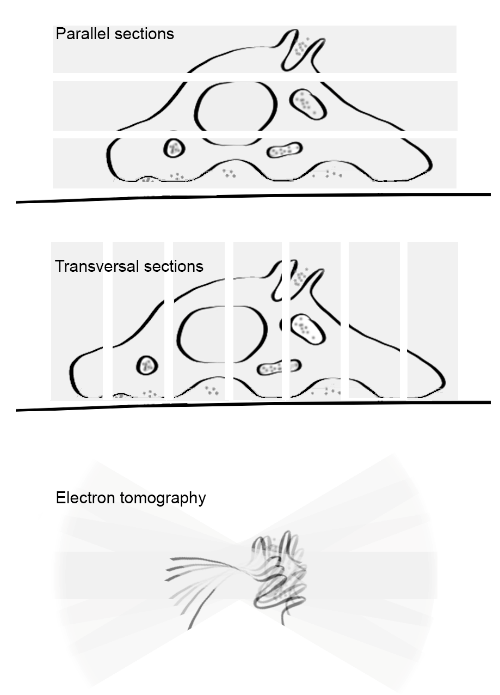

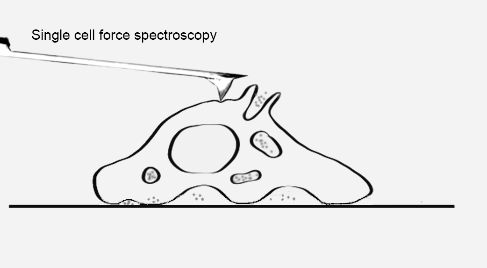

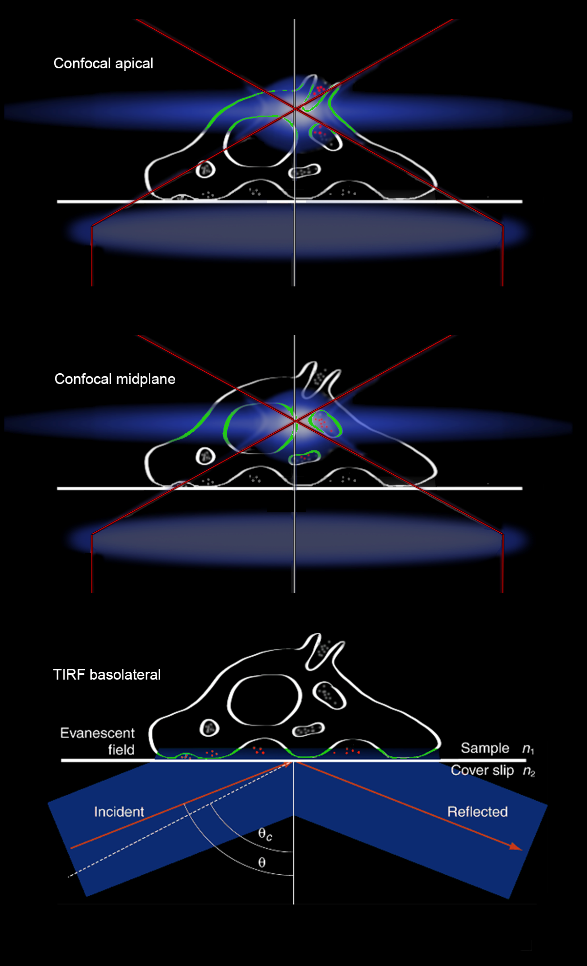


**C**

**A**

**B**

**D**

**Fig. S1.** Schematic representation of microscopy techniques. **A:** Single cell force spectroscopy. A tip representing a particle is lowered onto the cell, kept for a given amount of time, retracted, and the adhesion measured (Figure 1A-C). **B:** Electron microscopy of parallel sections of the epithelial cell layer (top, used for the electron tomogram in Fig. 2) and cross sections (Fig. 5A, C and 6L). **C:** Electron tomography: micrographs are recorded from the same section at multiple tilt angles with respect to the electron beam and the three-dimensional structure computed (Fig. 2A-C). **D:** Top: a sketch of an optical confocal section of the apical side of the epithelial cell culture (e.g. Fig. 4 A-F). Membranes fluoresce green where illuminated and particles are red. Middle: an optical confocal section of the midplane of the epithelial cell (e.g. Fig. 4 J-L). Bottom: The sketch illustrates TIRF imaging at an illumination depth <100 nm (e.g. Fig. 5 B,D,F, 6 A-F, I-K). The plasma membrane appears green where in direct contact with the coverslip (adhesion plaques) as well as for the fringes of these regions. In-between adhesion plaques, the plasma membrane does not get illuminated and the region appears black. Red fluorescent particles in proximity to the cover slip too, are illuminated at this shallow TIRF angle. This mostly happens within the black regions. In this case, particles are incontrovertibly outside of the cell. On the left of the sketched cell, green- and red fluorescence coincide where both structures are within the shallow evanescent field. In these instances, the resolution of the light microscope is insufficient to distinguish whether these particles are inside or outside of the cell. We therefore excluded these regions from quantification of export. Such situations are not hypothetical and readily observed by TEM. For example, Fig. 6L shows particles very close to the basolateral membrane inside the cell and Fig. 5A, and C particles outside the cell but the membrane still very close.


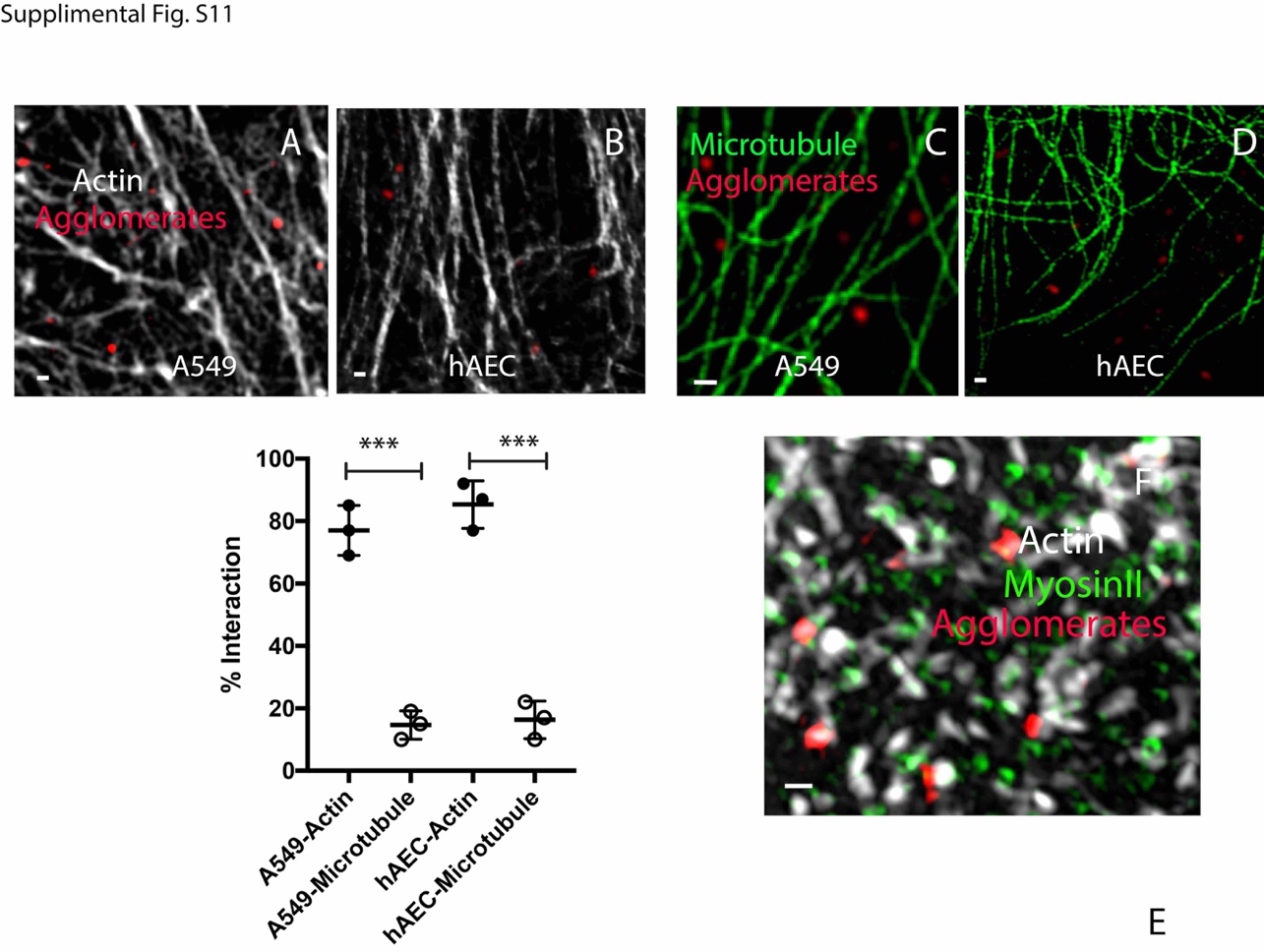


**Fig. S2.** **A-D:** Roles of actin and microtubules in particle transport. Structured Illumination microcopy of protein associations. Nanoparticles in close proximity to F-actin bundles in A549 cells (A) and in hAECs (B). **C-D:** Nanoparticle agglomerates are not associated with microtubules in A549 (C) and in hAECs (D). **E:** Proximity analysis shows in A549 78 ± 7.9% nanoparticles are in close association to F-actin, whereas 13 ± 5.2% nanoparticles are in proximity to microtubules, in hAECs 84±8.7% nanoparticle agglomerates are in close association of actin, whereas 16± 6.3 % nanoparticles were close to microtubules. Data are mean ± SD, n=5, ***p<0.001. **F:** SIM image shows association of the agglomerates to actin-myosin in hAEC, Scale bars: A-D, F 1 μm.


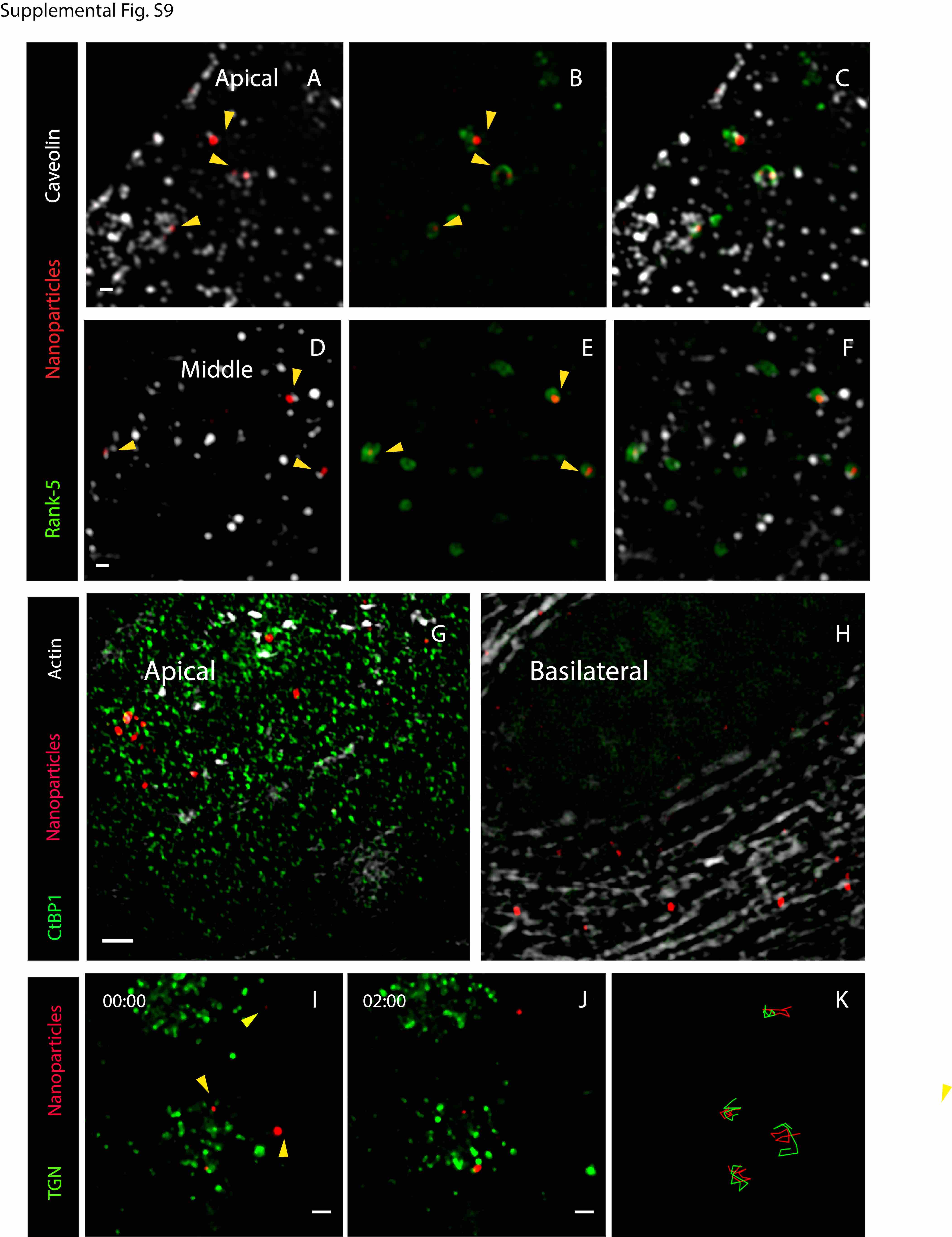


**Fig. S3.** Nanoparticle interactions with caveolin-1 and Rank-5 in A549. These data supplement that provided in Fig. 4 G-I using hAEC. Interactions of caveolin-1 and Rank-5 with nanoparticles in A549 cell were examined using super resolution microscopy. A slice at the apical surface is shown in (**A-C**) and a middle slice is shown in (**D-F**). Like hAECs, the particle compartments are associated with both caveolin-1 and Rank-5 in A549 cells. Scale bars: A-F 1 µm


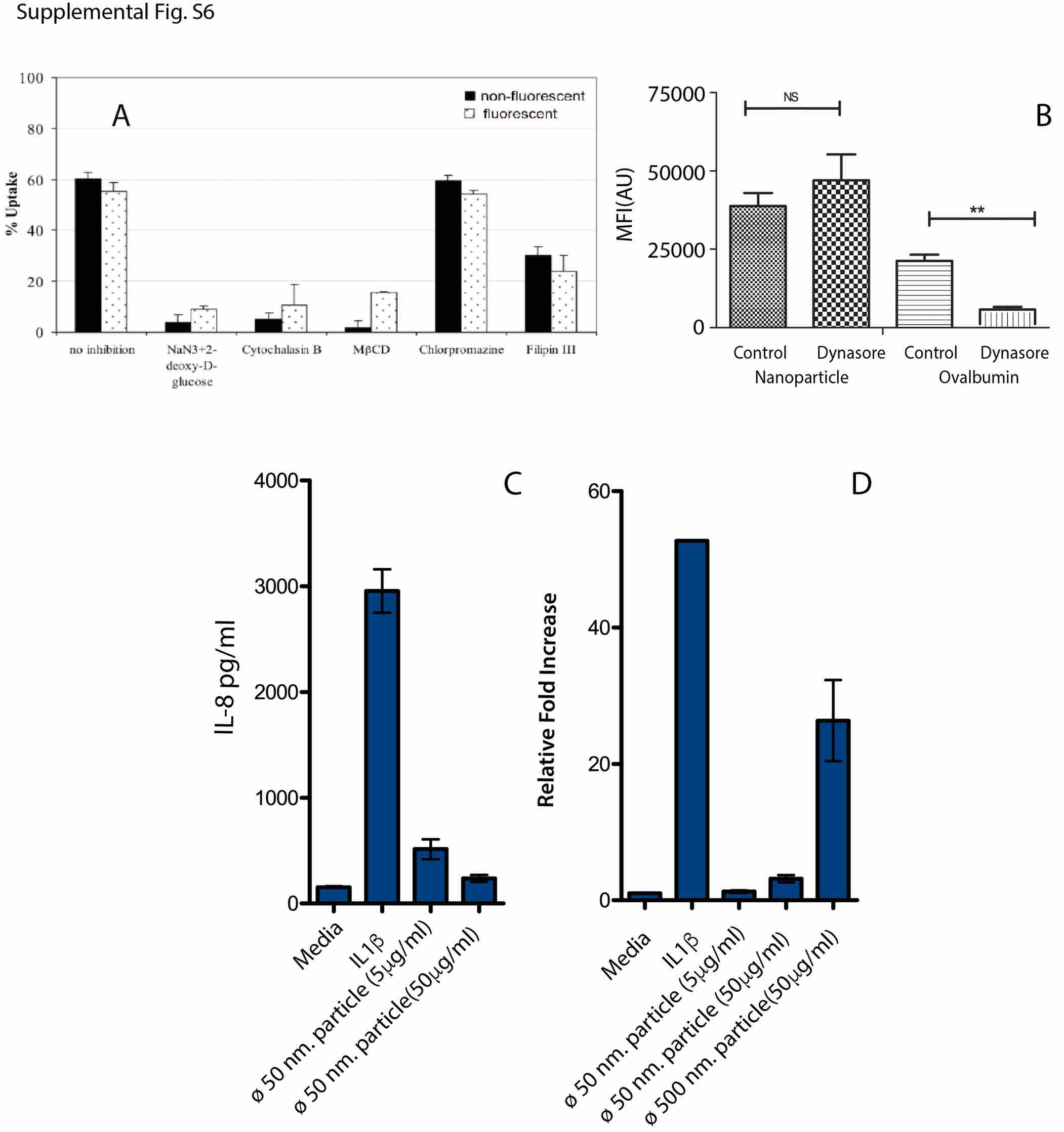


**Fig. S4.** Supplemental information for nanoparticle uptake by A549 cells. Effect of dynasore on nanoparticle uptake by A549. Dynasore did not inhibit red fluorescent nanoparticle uptake, whereas uptake of fluorescently labeled ovalbumin was inhibited. Data are mean ± SD, n=3, **p<0.01.

**
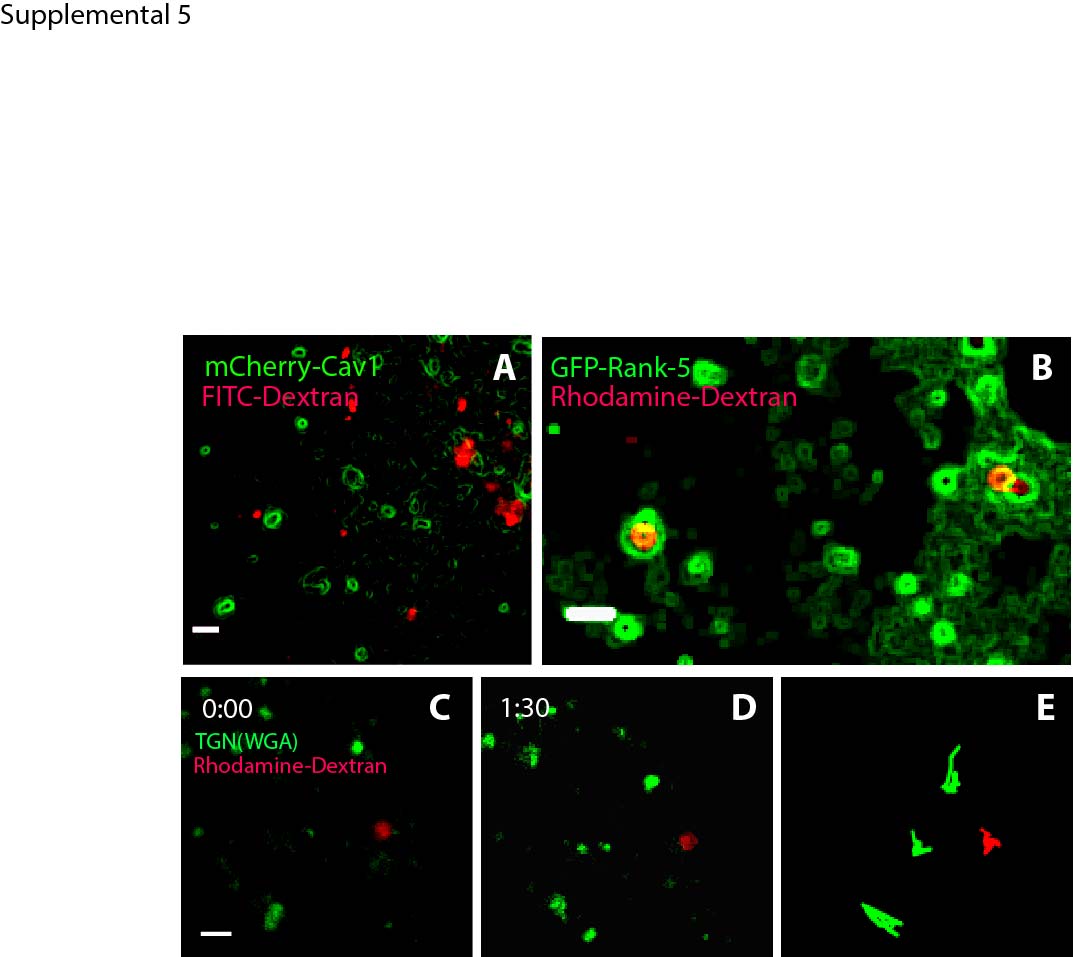
**

**Fig. S5. A**: As a negative control for classical macropinocytosis, FITC-dextran 90,000 daltons (pseudo-colored in red) did not overlap with the caveolin compartment during live cell imaging. **B:** In contrast, Rank-5, a marker for macropinocytosis covered the macropinosomes with Rhodamine-dextran. **C-E:** The TGN compartments did not move along with rhodamine-dextran. Figure shows two representative time points (G, H) and the corresponding trajectories of rhodamine-dextran and TGN (E). Scale bars: E-I 2 µm


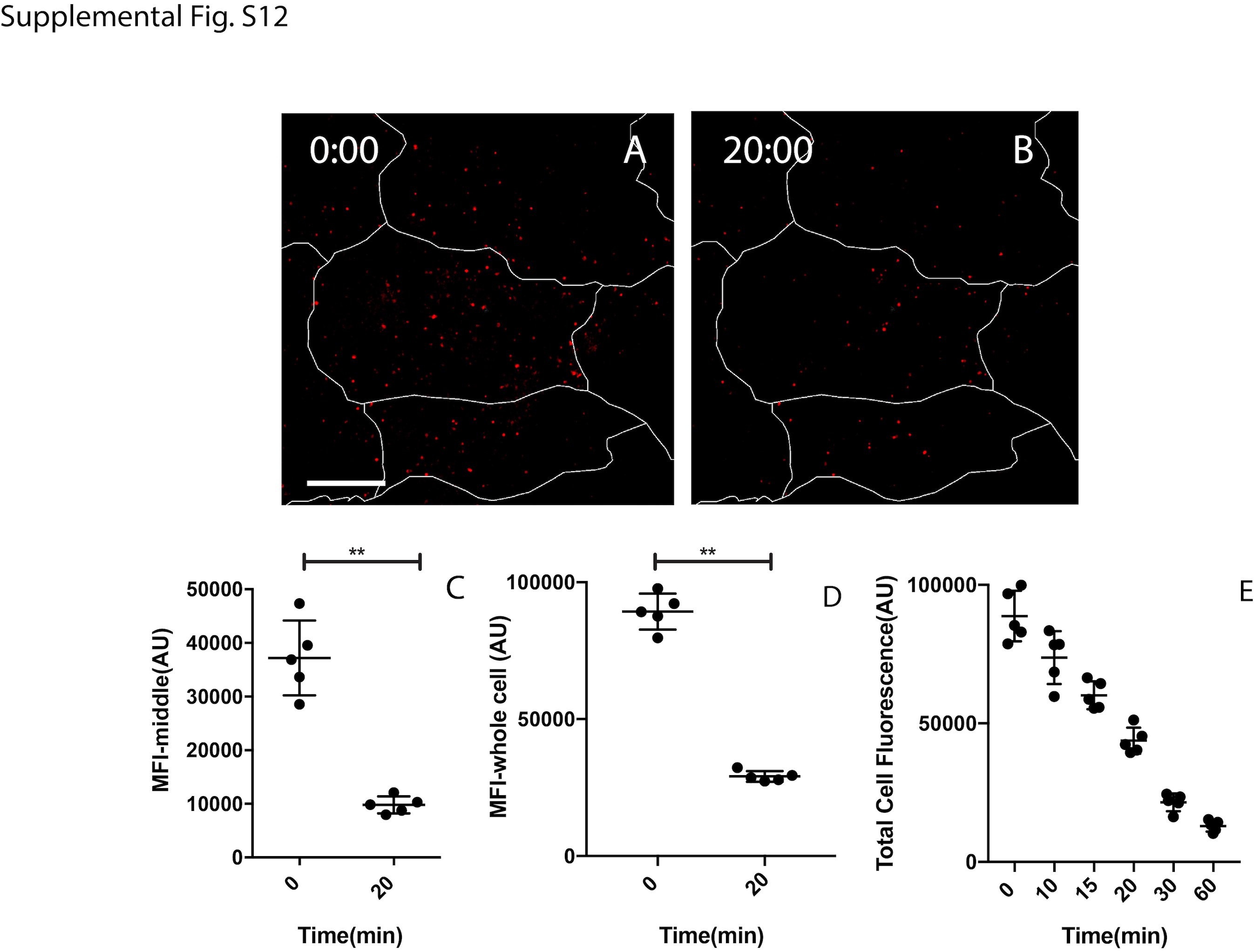


**Fig. S6.** Supplemental information to Fig 5. Evidence of exocytosis by confocal imaging. Nanoparticles were added to a confluent monolayer of hAECs and incubated for 1h after which time the remainder of the particles in the media were washed away. **A-B**: From there on, we used confocal imaging to determine the mean fluorescence intensity over time. **C-D:** We quantified fluorescence from either (C) the mid-plane of the cell or (D) measured total fluorescence from a maximum intensity projection of the all the optical sections. Both measurement strategies showed a similar reduction in nanoparticles over time due to particle export. **E:** Alternatively, dishes of confluent monolayer of hAECs were treated with particles as above. Cells were harvested after 10, 15, 20, 30 & 60 mins by trypsin, washed then lysed and the fluorescence read on a plate reader. Consistent with confocal images, these data showed a similar reduction of the relative fluorescence over time. Data are mean ± SD, n=5, **p<0.01. Scale bars: A-B 5 µm.


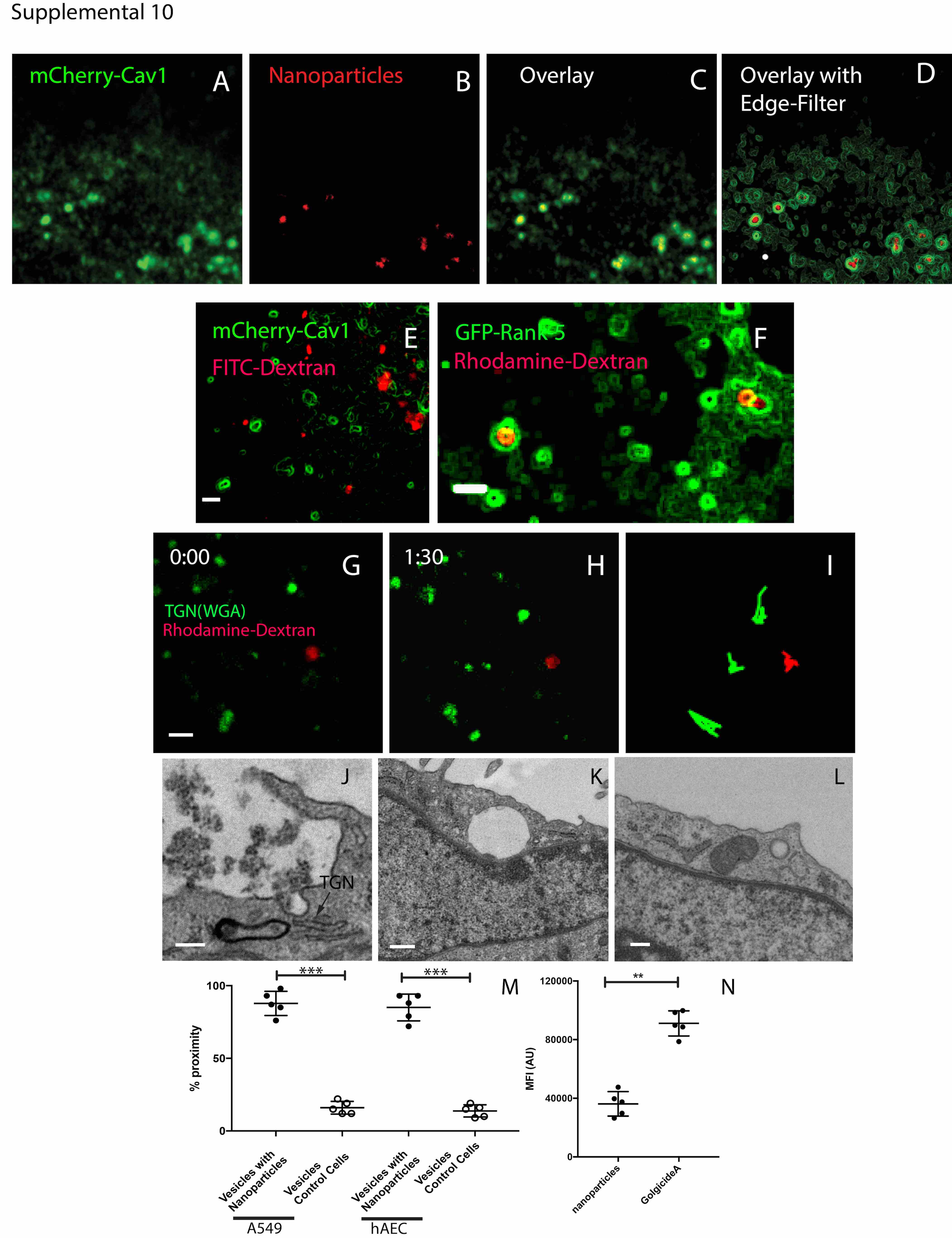


**Fig. S7.** Disruption of Golgi by golgicide A does not affect uptake but blocks the export of the particles. A confluent monolayer of hAECs was pretreated with golgicide A (30 min), incubated with nanoparticles for 1h and washed. Particles within the cell were quantified from the mean fluorescence intensity of a mid-plane after one hour. Data are mean ± SD, n=5, **p<0.01.

**Supplemental Movie legends:**

**Movie S1**, supplementary to Fig. 2 E: Confocal live cell imaging of eGFP lifeact-transfected A549 at the apical side of the cell. Remodeling of actin fibers leads to the internalization of the agglomerates (red). Note that often, a prominent fiber develops at the trailing end of the moving agglomerate (e.g. 08:00 to 8:30 [min:sec]), suggesting that the compartment may be pushed, rather than pulled. Free agglomerates in solution float by (e.g. 04:36 to 04:48). Scale bar 2 µm.

**Movie S2**, supplementary to Fig. 4 D-F: A z-stack of confocal sections displayed from the apical surface down to 2.4 microns into the cell. GFP-Rank-5 (green) is displayed after applying an edge filter. At the cell surface in the initial adhesion phase, the red-fluorescent nanoparticle agglomerate is not associated with Rank-5. Inside the cell, the particle compartments are lined by Rank-5. Scale bar 2 µm.

**Movie S3**, supplementary to Fig. 4 D-F: A time-lapse of confocal live cell images of a GFP-Rank-5 transfected cell, incubated with red-fluorescent nanoparticles. The movie shows a time-lapse of maximum intensity projections of a cell from apical to 2.4 micron into the cell. The kymographs for two agglomerates inside the cell show the rapid movement of the particle compartment at this point. The agglomerate at the apical surface of the cell was not surrounded by Rank-5 and did barely move. Scale bar 2 µm.

**Movie S4**, supplementary to Fig 4 J-L: live cell imaging of nanoparticles (red) and TGN (green) in an hAEC (left pane without and right pane with kymograph). The video was recorded from a single confocal plane of about 0.4µm depth in the middle of the cell. Four out of five particle compartments move in a correlated manner with a TGN granule. Scale bar 2 µm, imaging plane.

**Movie S5**, supplementary to Fig 5: Live cell TIRF imaging (illumination depth: 100 nm) of red-fluorescent particles entering the pericellular space between coverslip and the basolateral side of plasma membrane-labeled hAECs (Alexa 488-WGA). The shallow TIRF illumination causes only the adhesion patches of the plasma membrane to fluoresce green (see also Fig. 5). The remainder of the basolateral membrane is detached from the coverslip and outside of the illumination sheet (dark regions). Hence, when red fluorescent agglomerates appear in the dark regions, they are beneath the basolateral plasma membrane and thus exocytosed. Particles appeared in the pericellular place in-between WGA patches as early as 10 minutes after the addition of nanoparticles. The numbers of particles increased progressively in a time dependent manner Scale bar 2 µm.

**Movie S6**, supplementary to Fig. 6 A-C: Live cell TIRF imaging (illumination depth: < 250 nm) of the basolateral plane of A549, transfected with GFP-Rab6A. Left pane without, right pane with kymograph. Rab6A is a trans Golgi-associated (TGN) exocytosis marker. The video shows the correlated movement of the particle compartment (red) and its associated TGN granule (green) for two pairs. The structures are sometimes seen side by side with minimal overlap (e.g. @00:25 [min:sec] for the pair on the left and @00:50 for the pair on the right) or on top of each other (@00:50 [min:sec] for the pair on the left and @01:20 for the pair on the right), consistent with two linked globes that rotate and translate as an entity. As well, globes appear to increase and decrease in size, likely as a result of becoming more or less immersed in the illumination plane while moving across the cell. Scale bar 2 µm.

**Movie S7**, supplementary to Fig. 6 D-F: Live cell TIRF imaging (illumination depth: < 250 nm) of the basolateral plane of A549, transfected with GFP-Rab6A in presence of the myosin II inhibitor blebbistatin. The video shows minimal movement of Rab6A as well as particle compartment, indicating myosin II plays a role in particle transport. Scale bar 2 µm.

**Movie S8**, supplemental to Fig. 6 I-K: Live cell TIRF imaging (illumination depth: < 250 nm) of particles with respect to actin fibers near the basolateral surface of the A549 cells. The yellow track indicates the movement of one large agglomerate at the basolateral side as it follows an actin fiber. Note that particles can only be tracked as long as they stay within the narrow illumination sheet afforded by TIRF. Other particles appear briefly and then disappear, as they dip in and out of the illumination sheet. Scale bar 1 µm.
